# Mutations in LRRK2 amplify cell-to-cell protein aggregate propagation: a hypothesis

**DOI:** 10.1007/s00401-018-1838-7

**Published:** 2018-03-29

**Authors:** Patrick A. Lewis

**Affiliations:** 1 School of Pharmacy, University of Reading, Whiteknights, Reading RG6 6AP UK; 2 Department of Molecular Neuroscience, UCL Institute of Neurology, Queen Square, London, WC1N 3BG UK

## Introduction

Mutations in the *LRRK2* gene are the most common genetic cause of Parkinson’s disease (PD) [6]. Despite over a decade of research, the function of leucine-rich repeat kinase 2 (LRRK2), the multidomain, dual activity enzyme product of the *LRRK2* gene, remains enigmatic—as does the nature of the dysfunction caused by mutations that leads to degeneration in the human brain.

**Fig. 1:**
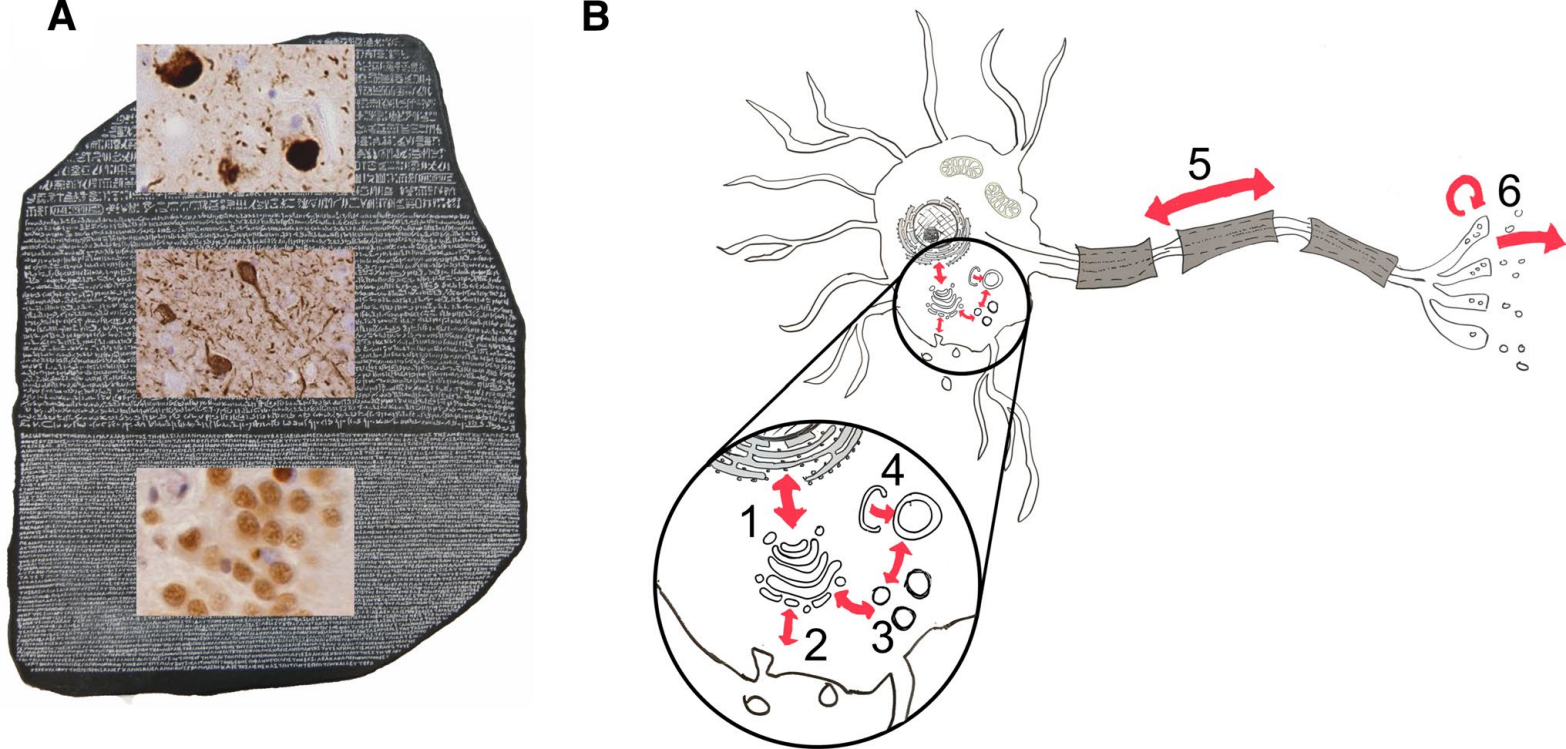
**a** LRRK2 and pleomorphic pathology: a Rosetta stone for neurodegeneration. LRRK2 mutation carriers present with alpha-synuclein pathology (top panel, Hieroglyphs), tau pathology (middle panel, Demotic script), and TDP-43 pathology (bottom panel, Greek). **b** The reported cellular functions of LRRK2 with potential relevance to protein aggregate transmission, modeled in an archetypal neuron. (1) Golgi/endoplasmic reticulum shuttling (2) endocytosis/exocytosis (3) Golgi/endosome trafficking (4) autophagy/lysosomal function (5) axonal transport (6) synaptic vesicle function

*LRRK2* parkinsonism is characterized by a clinical presentation that is almost indistinguishable from idiopathic Parkinson’s, and the *LRRK2* locus is associated with genome-wide associated risk for idiopathic Parkinson’s linked to the promoter region of the gene [6]. These attributes, coupled to LRRK2 possessing two potentially druggable enzymatic activities (a kinase and a GTPase), have led to LRRK2 being the focus of significant drug discovery efforts—with LRRK2 kinase inhibitors currently undergoing phase I clinical trials. Intriguingly, one aspect of LRRK2 pathobiology stands out as being distinctive in the field of neurodegeneration—pleomorphic neuropathology [13]. Although the predominant neuropathological lesion in the brains of patients with *LRRK2* parkinsonism is the Lewy body, a significant minority of cases present with other forms of protein aggregate pathology. These include neurofibrillary tangles consisting of aggregated tau and consistent with a diagnosis of progressive supranuclear palsy (PSP), TDP-43 positive inclusions similar to those observed in frontal–temporal dementia (FTD) and amyotrophic lateral sclerosis (ALS), and glial cytoplasmic inclusions normally associated with multiple system atrophy (MSA) [9]. There is no obvious pattern as to the development of these pathologies, with individuals from the same kindred with the same mutation and almost indistinguishable clinical presentation displaying divergent neuropathology. Mutations in LRRK2, therefore, provide a compelling example of a genetically defined disorder where the spatiotemporal pattern of neuronal degeneration is disengaged from the nature and character of the protein aggregates associated with it. This uncoupling of pathology and etiology in LRRK2 cases has fundamental implications for the way in which we conceptualize and categorize the neurodegenerative process. Indeed, this has led to LRRK2 being proposed as a Rosetta stone for neurodegeneration [1]. The implication of this being that by deciphering the function/s of LRRK2 determining these divergent pathologies, we will be able to translate between the pathological processes that span dementia, ALS, MSA, PSP and PD—just as the Rosetta stone allowed translation between Egyptian hieroglyphics, Demotic script and Greek (Fig. 1a).

Directly relevant to this phenomenon, there is a substantial body of evidence linking the pathological propagation of misfolded protein aggregates to neurodegeneration [3]. For Parkinson’s disease, data from human fetal transplants demonstrating that Lewy body pathology can spread to naïve cells, and animal models provide evidence that pre-formed aggregates of alpha-synuclein can propagate through the rodent brain support a role for protein aggregate spread in the disease process. It is, however, important to note that these data (or at least the interpretation of these data) remain controversial [12].

To explain the pleomorphic pathology observed in *LRRK2* mutation carriers, I propose that LRRK2 is involved in the regulation of cell-to-cell protein aggregate spreading and propagation. This hypothesis posits that the normal function of LRRK2 has a direct influence on the ability of protein aggregates to transfer between cells, acting to repress propagation, and that coding mutations in LRRK2 act as a gain of function to modify this physiological role. The cellular consequence of mutations in LRRK2 is, therefore, an amplification of protein templating and concomitant cytotoxicity leading to neurodegeneration and disease progression. A key element of this hypothesis is that the role that LRRK2 plays in the regulation of protein aggregate propagation is autonomous of the protein constituents of these aggregates. An initial stochastic misfolding event involving alpha-synuclein, therefore, would be amplified by the presence of a mutation in LRRK2 leading to Lewy body or glial cytoplasmic inclusion pathology. Were the initial misfolding event to involve tau, however, this would lead to the propagation of neurofibrillary tangle pathology—and likewise for TDP-43. This hypothesis provides a potential explanation for the puzzle of pleomorphic pathology.

## Evidence consistent with a role for LRRK2 in protein aggregation and propagation

Given the centrality of protein aggregation to our current understanding of neurodegeneration, it is unsurprising that multiple studies have sought to examine a link between LRRK2 and the formation/propagation of protein aggregates. The majority of these investigations have focused on alpha-synuclein, studying the nature of protein aggregation and how LRRK2 can influence this. In murine models for LRRK2 and alpha-synuclein dysfunction, several studies have shown that the nature and frequency of alpha-synuclein aggregates can be modulated by knockout of the *LRRK2* gene [4, 10], although there are counter examples from the literature [2]. Experiments using primary cells to model LRRK2 dysfunction have highlighted that the G2019S mutation (the most common coding mutation in LRRK2) can alter alpha-synuclein aggregation, and that this can be reversed using specific inhibitors of LRRK2 function [8, 11]. These experimental data do not directly examine a role for LRRK2 in the regulation of protein templating; however, a recent study in a mouse model for tau dysfunction suggested that LRRK2 could be acting to facilitate the spread of tau in the brain [5]. The putative relationship between LRRK2 and TDP-43 aggregation has not been examined at a molecular level in any detail.

## Functional links to LRRK2 biology

LRRK2 has been implicated in a wide range of cellular processes, however, there is increasing evidence linking it to the regulation of vesicular transport and catabolic processes in the cell [7]. In particular, LRRK2 has been linked to endosomal trafficking and the function of the autophagy/lysosomal pathway, axonal transport, and synaptic function (including endocytosis) (Fig. 1b). Each of these processes have the potential to be important determinants of protein aggregation or propagation, with the autophagy/lysosomal system critical for proteostasis and the catabolism of nascent protein aggregates; and endosomal trafficking, axonal transport, synaptic function and exosomes playing roles in the passage of aggregates around and between cells. Which of these is important for the etiology of LRRK2 Parkinson’s disease, and (in the context of this hypothesis) protein aggregate propagation, is not yet clear.

## Experimental testing

There are a number of experiments that would test whether LRRK2 has a conserved role in the regulation of cell-to-cell propagation of protein aggregates involved in neurodegeneration. Cell and animal systems have been developed to model the propagation of protein aggregates in the brain, including alpha-synuclein, tau and TDP-43. These provide a platform for investigating how LRRK2 biology intersects with protein aggregate spreading across the range of pathologies observed in LRRK2 neurodegenerative disease. By combining these model systems with genetic models for LRRK2 (for example knockin coding mutations associated with disease, or knockout of the *LRRK2* gene), alongside highly specific brain-penetrant inhibitors of LRRK2 kinase function, it will be possible to directly test this hypothesis. If correct, the prediction would be that disease-associated mutations in LRRK2 act to perpetuate and amplify protein aggregate propagation, whereas inhibition of LRRK2 kinase activity or loss of function would act to reduce aggregate spread.

## Discussion

A number of questions arise from this hypothesis and the existing LRRK2 literature.

First, it is not yet clear which cell type within the brain drives the pathological associations between LRRK2 and the degeneration of dopaminergic neurons in the Substantia nigra pars compacta. Expression of LRRK2 is low in these cells, and there is increasing evidence for a functional role for LRRK2 in glial cell populations within the brain. A better understanding of the cellular expression of LRRK2, coupled with experiments testing whether the cytotoxic impact of mutations in LRRK2 is cell autonomous, is, therefore, a key area for research over the coming years.

Second, if it is the case that LRRK2 plays a generic role in the propagation of protein aggregates in the human brain, why is it that individuals with mutations in *LRRK2* predominantly develop Parkinson’s rather than FTD, PSP or ALS? Although there are case reports for *LRRK2* cases with a clinical diagnosis of PSP, these are rare. This issue sits at the heart of our understanding of neurodegeneration, and the issue of selective neuronal vulnerability in distinct neurological disorders. While this is an unresolved issue across the whole spectrum of neurodegenerative disease, it has been suggested that a combination of the expression of specific combinations of gene, coupled to individual neuronal characteristics (such as pacemaker activity), may result in differential vulnerability, and based upon this it is likely that a similar combination of factors determines selective neuronal vulnerability in *LRRK2* PD.

A third issue is why the pathological aggregates observed in *LRRK2* mutation carriers are limited to those consisting of alpha-synuclein, tau and TDP-43. If there is a conserved role for LRRK2 in the transmission of protein aggregates, why do patients not present with aggregation of amyloid beta or the accumulation of misfolded prion protein? The answer to this may lie in the predominantly intracellular nature of alpha-synuclein, tau and TDP-43 aggregates, and again this could be tested using models for amyloid beta or prion protein propagation.

In summary, the origin of the pleomorphic pathology observed in *LRRK2* mutation carriers presenting with Parkinson’s remains a mystery. As outlined above, the hypothesis that mutations in the *LRRK2* gene drive an increased propensity for pathologically associated protein aggregates to convert from isolated stochastic misfolding events to widespread accumulation and cytotoxicity provides a plausible explanation for this phenomenon.
